# Periprocedural myocardial injury according to optical characteristics of neointima and treatment modality of in-stent restenosis

**DOI:** 10.1007/s00392-022-02024-z

**Published:** 2022-04-27

**Authors:** Nejva Nano, Alp Aytekin, Gjin Ndrepepa, Masaru Seguchi, Jola Bresha, Hector Alfonso Alvarez Covarrubias, Philipp Nicol, Tobias Lenz, Shqipdona Lahu, Senta Gewalt, Felix Voll, Tobias Rheude, Jens Wiebe, Heribert Schunkert, Sebastian Kufner, Salvatore Cassese, Michael Joner, Adnan Kastrati, Erion Xhepa

**Affiliations:** 1 Klinik Für Herz- Und Kreislauferkrankungen, Deutsches Herzzentrum München, Technische Universität München, Lazarettstrasse 36, 80636 Munich, Germany; 2 Medizinische Klinik Und Poliklinik Innere Medizin I, Klinikum Rechts Der Isar, Technische Universität München, Munich, Germany; 3 DZHK (German Centre for Cardiovascular Research), Partner Site Munich Heart Alliance, Munich, Germany

**Keywords:** Drug-eluting balloon, Drug-eluting stent, In-stent restenosis, Neointimal characterization, Optical coherence tomography, Periprocedural myocardial injury

## Abstract

**Aims:**

Aim of the present study was to investigate the impact of increasing neointimal inhomogeneity and neoatherosclerosis as well as of treatment modality of in-stent restenosis (ISR) on the occurrence of periprocedural myocardial injury (PMI).

**Methods and results:**

Patients with normal or stable/falling increased baseline high-sensitivity troponin T (hs-cTnT) undergoing intravascular optical coherence tomography (OCT) and subsequent percutaneous coronary intervention (PCI) of ISR by means of drug-coated balloon (DCB) or drug-eluting stent (DES) were included. Overall, 128 patients were subdivided into low (*n* = 64) and high (*n* = 64) inhomogeneity groups, based on the median of distribution of non-homogeneous quadrants. No significant between-group differences were detected in terms of hs-cTnT changes (28.0 [12.0–65.8] vs. 25.5 [9.8–65.0] ng/L; *p* = 0.355), or the incidence of major PMI (31.2 vs. 31.2%; *p* = 1.000). Similarly, no differences were observed between DCB- and DES-treated groups in terms of hs-cTn changes (27.0 [10.0–64.0] vs. 28.0 [11.0–73.0] ng/L; p = 0.795), or the incidence of major PMI (28.9 vs. 35.6%; *p* = 0.566). Additionally, no significant interaction was present between optical neointimal characteristics and treatment modality in terms of changes in hs-cTnT (*P*_int_ = 0.432). No significant differences in PMI occurrence were observed between low and high neoatherosclerosis subgroups.

**Conclusions:**

In patients undergoing PCI for ISR, there was no association between increasing neointimal inhomogeneity, or increasing expression of neoatherosclerotic changes and occurrence of PMI. PMI occurrence was not influenced by the treatment modality (DCB vs. DES) of ISR lesions, a finding that supports the safety of DCB treatment for ISR.

**Graphical abstract:**

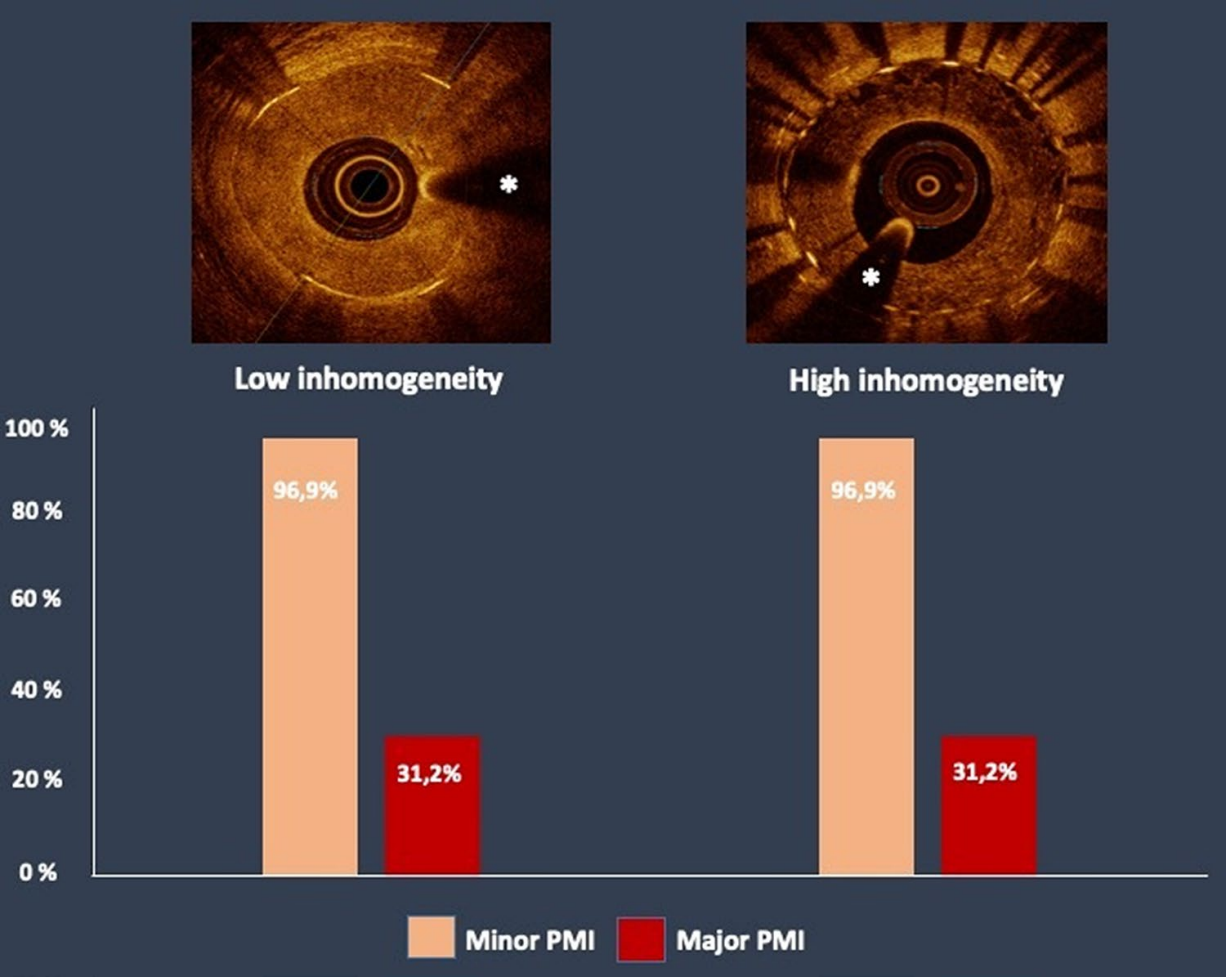

**Supplementary Information:**

The online version contains supplementary material available at 10.1007/s00392-022-02024-z.

## Introduction

Although persistent iterations in drug-eluting stent (DES) technology have markedly reduced the occurrence of in-stent restenosis (ISR), such clinical entity still represents a significant burden for patients undergoing percutaneous coronary intervention (PCI) [1]. A recent nationwide registry in the United States found that ISR-PCI represented nearly 10% of the total PCI procedures, with approximately 25% of patients presenting with acute myocardial infarction (MI) [2].

Based on the currently available evidence, European guidelines recommend either drug-coated balloon (DCB) angioplasty or DES implantation as treatment options for ISR [3]. Periprocedural myocardial injury (PMI) represents an intrinsic risk of PCI, which has been reported in a significant proportion of patients undergoing PCI for stable coronary artery disease (CAD) [4–9]. While large periprocedural myonecrosis and MI almost invariably correlate with readily recognizable complications at angiography, PMI is frequently observed following otherwise uneventful PCI procedures. The prognostic implications of PMI remain controversial and data from available studies have reported discordant results [4, 5, 7–10]. Several imaging studies in the setting of native vessel PCI have shown a relationship between PMI and presence of lipid-rich plaques or large necrotic cores, which may predispose to peripheral embolization with resulting microvascular obstruction [11, 12]. A very limited number of studies have investigated the relationship between neointimal tissue characteristics and PMI following ISR-PCI [13, 14]. The use of intravascular optical coherence tomography (OCT) allows a detailed analysis and classification of neointimal tissue in varying patterns that correlate with different histological substrates [15, 16].

Beside neointimal tissue characteristics, treatment modality may represent an additional mechanism influencing PMI occurrence in the setting of ISR-PCI. Indeed, in order to improve its solubility and prevent clumping of particles on the DCB surface, paclitaxel is mixed with a hydrophilic excipient and, as occasionally observed in preclinical studies, DCB angioplasty may be associated with a risk of distal embolization of particulate balloon coating, consisting of antirestenotic drug and excipient [17].

Against this background, the aim of the present study was to investigate the impact of increasing neointimal inhomogeneity and neoatherosclerosis, as well as of treatment modality (DCB vs. DES) on the occurrence of PMI.

## Methods

### Patients, procedures and definitions

Patients undergoing intravascular OCT and subsequent DCB angioplasty or DES implantation for ISR at the Department of Cardiology of the German Heart Centre Munich were included. In order to attribute with certainty increases in cardiac biomarkers to the PCI procedure, according to the fourth universal definition of myocardial infarction (UDMI) [6], only patients with normal baseline high-sensitivity troponin T (hs-cTnT) (≤ 99th percentile upper reference limit [URL]) or with stable/falling increased baseline values (≥ 99th percentile URL) were included. PMI was defined according to the criteria of the 4th UDMI and of a recent ESC/EAPCI consensus document [9]. In patients with normal baseline values, an increase of hs-cTnT > 99th percentile URL was considered as minor PMI and an increase of hs-cTnT > 5 × 99th percentile URL was considered as major PMI. In patients with stable/falling increased baseline hs-cTnT, the diagnosis of PMI required in addition a rise of hs-cTnT > 20% of the baseline value. Informed consent was obtained prior to each procedure. Clinical follow-up was performed by office visit, phone contact or structured follow-up letter.

### Angiographic and OCT image acquisition and analysis

**Fig. 1:**
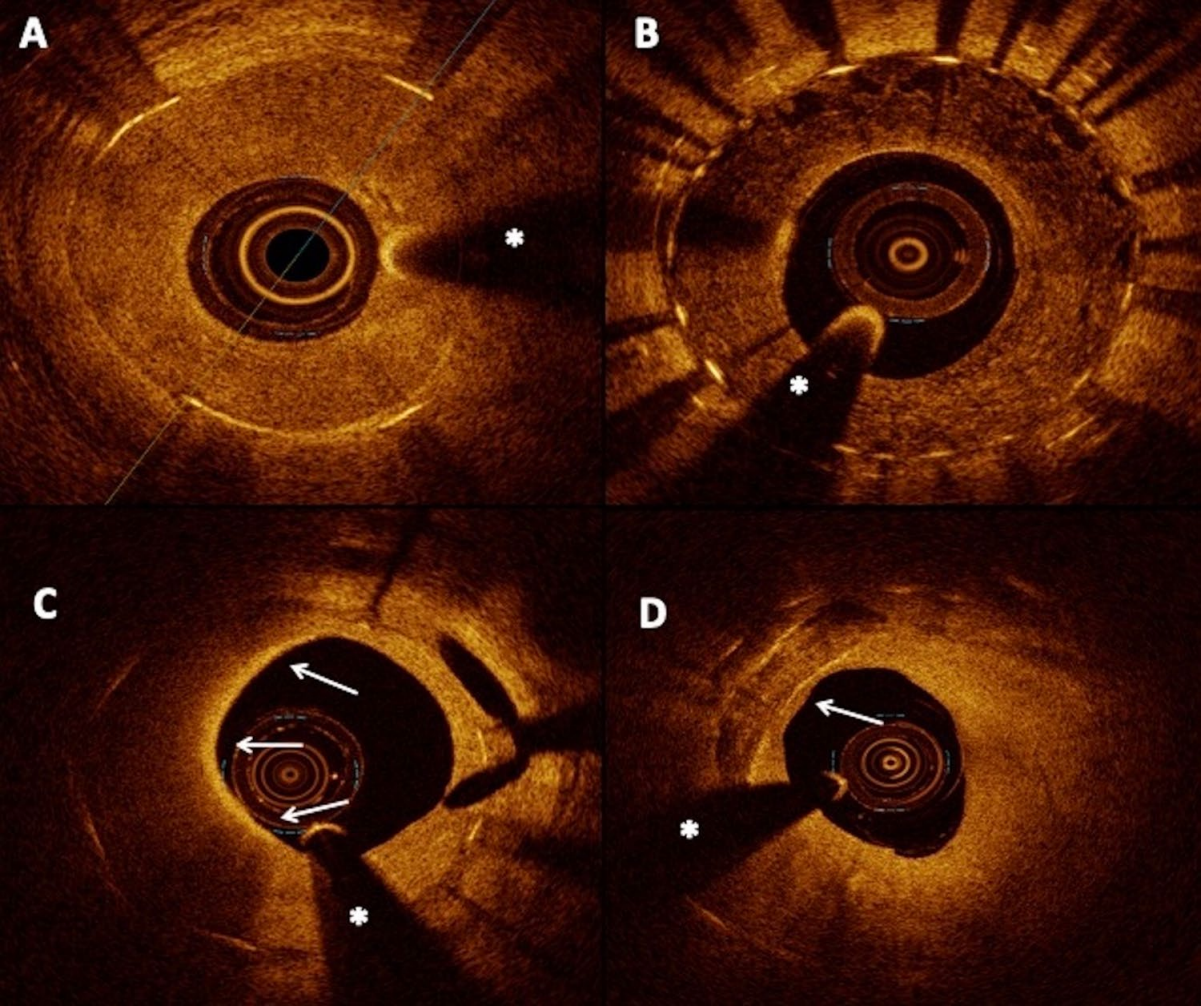
Images of optical coherence tomography findings in patients presenting with in-stent restenosis. **A** Homogeneous neointimal pattern. **B** Heterogeneous neointimal pattern. **C** Neoatherosclerosis with macrophage infiltration involving a 180° neointimal arc (arrows). **D** Neointimal calcification (arrow) * = guidewire artifact

Baseline and post-procedural angiograms and raw data of OCT image acquisitions were recorded and assessed off-line in a core laboratory (ISAResearch Center, Munich, Germany). Quadrant-based neointimal characterization was performed at the frame displaying the maximal % area stenosis and the five preceding and following analyzed frames [15, 18]. Neointimal tissue was categorized as homogeneous or inhomogeneous, the latter category including heterogeneous, layered or neoatherosclerosis quadrants. Atherosclerotic changes of the neointima were defined by the presence of one or more of the following: macrophage infiltration, lipid-laden tissue within the stent or neointimal calcification (Fig. 1). Further details and definitions regarding angiographic and OCT analysis are provided in the Supplementary Appendix.

To investigate the impact of an increasing presence of inhomogeneous quadrants on PMI, the study population was divided in low and high inhomogeneity groups, based on the median of distribution of non-homogeneous quadrants; the high inhomogeneity patient subgroup was further classified in low and high neoatherosclerosis subgroups.

### Biochemical measurements

Blood samples for hs-cTnT measurements were collected in tubes containing lithium‐heparin anticoagulant at the time of admission, 3–6 h after PCI, at 6 h intervals in case of rising values, and on a daily basis thereafter. The plasma concentration of hs‐cTnT was measured using a high‐sensitivity assay on a Cobas e411 immunoanalyser based on electrochemiluminescence technology (Roche Diagnostics, Rotkreuz, Switzerland). The limit of blank for this assay—the concentration below which analyte-free samples are found with a probability of 95%—is ≤ 3 ng/L. The functional sensitivity—the lowest analyte concentration that can be reproducibly measured with a coefficient of variation ≤ 10%—is ≤ 13 ng/L. The 99th URL is 14 ng/L. Baseline and peak post-procedural hs-cTnT were used for the current analysis. Other biochemical parameters were measured using standard laboratory methods.

### Statistical analysis

Continuous data are presented as mean ± SD or median (25th–75th percentiles) depending on the distribution pattern of the variable. Categorical data are presented as absolute and relative frequencies (%). Differences between groups were compared using the Student’s *t* test or the Wilcoxon rank sum test for continuous variables and the Pearson *χ*^2^ test (or Fischer’s exact test where any expected cell count of the contingency table was < 5) for categorical variables. To account for the clustered nature of the data, a linear mixed model was used for the analysis of OCT data. The model contained a fixed-effects term (neointimal pattern) and a random intercept as random-effects term for patient in case of frame-level analysis and as nested random-effects term for patient and frame for strut-level analysis. A multivariable model including baseline clinical, angiographic and procedural characteristics in addition to the optical pattern of neointima was performed to evaluate the potential independent impact of neointimal pattern of ISR on changes in hs-cTnT. An interaction test was conducted in order to assess whether the relation between optical characteristics of neointima and PMI occurrence is influenced by the treatment modality of ISR. Event-free survival was estimated by the Kaplan–Meier method for each clinical outcome. Hazard ratios (HR) with two-sided 95% confidence intervals (95%CI) were calculated using Cox proportional hazards models. All tests were two-sided and assessed at a significance level of 5%. Statistical analysis was performed using the R 3.6 Statistical Package (R Foundation for Statistical Computing, Vienna, Austria).

## Results

### Baseline clinical, angiographic and procedural characteristics

Overall, 128 patients were included, with one lesion being imaged/treated per patient. Based on the median of distribution of non-homogeneous quadrants, patients were divided into low (*n* = 64) and high (*n* = 64) inhomogeneity groups. Baseline clinical, angiographic and procedural characteristics according to neointimal tissue characterization are shown in Table 1 and Table 2. Besides target coronary vessel, no differences in terms of clinical, angiographic or procedural characteristics were observed between the groups. The underlying stent requiring repeat PCI due to presence of ISR was mostly represented by a DES. Treatment modality consisted of DES implantation in 45 (35.2%) and DCB angioplasty in 83 (64.8%) patients. Finally, no significant differences in terms of quantitative coronary analysis (QCA) parameters were observed between the groups. Supplementary Tables 1 and 2 report the clinical, angiographic and procedural characteristics according to treatment modality and Supplementary Tables 3 and 4 report the same characteristics according to extent of neoatherosclerosis in the subgroup of patients with high neointimal inhomogeneity.

**Table 1:** Clinical characteristics according to neointimal tissue characterization

	Low inhomogeneity *N* = 64	High inhomogeneity *N* = 64	*p* value
Age, years	66.8 ± 10.8	68.3 ± 8.9	0.380
Sex, male	10 (15.6)	13 (20.3)	0.645
Body mass index, kg/ m^2^	28.6 ± 3.8	28.4 ± 4.6	0.787
Current smoker	13 (20.3)	10 (15.6)	0.645
Ex-Smoker	20 (31.2)	23 (35.9)	0.708
Hypercholesterolemia	43 (67.2)	44 (68.8)	1.000
Arterial hypertension	61 (95.3)	63 (98.4)	0.619
Diabetes mellitus	29 (45.3)	28 (43.8)	1.000
Oral therapy	16 (25.0)	16 (25.0)	1.000
Insulin therapy	11 (17.2)	6 (9.4)	0.298
Previous coronary artery bypass surgery	10 (15.6)	12 (18.8)	0.815
Previous myocardial infarction	34 (53.1)	30 (46.9)	0.596
Clinical presentation			0.206
Silent Ischemia	16 (25.0)	14 (21.9)	
Stable Angina Pectoris	37 (57.8)	45 (70.3)	
Unstable Angina Pectoris	11 (17.2)	5 (7.8)	
Number of diseased coronary arteries			0.621
One vessel	6 (9.4)	8 (12.5)	
Two vessels	10 (15.6)	13 (20.3)	
Three vessels	48 (75.0)	43 (67.2)	
Multi-vessel disease	58 (90.6)	56 (87.5)	0.777
Left ventricular ejection fraction, %	52.7 ± 10.0	51.9 ± 10.3	0.786

Data are shown as counts (%) or mean ± SD (standard deviation)

**Table 2:** Angiographic and procedural characteristics according to neointimal tissue characterization

	Low inhomogeneity *N* = 64	High inhomogeneity *N* = 64	*P* value
Target vessel			0.033
Left main coronary artery	0 (0.0)	3 (4.7)	
Left anterior descending artery	30 (46.9)	30 (46.9)	
Left circumflex artery	11 (17.2)	19 (29.7)	
Right coronary artery	23 (35.9)	12 (18.8)	
Restenosis morphology			0.106
Focal margin	2 (3.1)	5 (7.8)	
Focal body	32 (50.0)	30 (46.9)	
Multifocal	10 (15.6)	2 (3.1)	
Diffuse intrastent	18 (28.1)	23 (35.9)	
Proliferative	1 (1.6)	1 (1.6)	
Complete occlusion	1 (1.6)	3 (4.7)	
Index Stent Interval, days	364 (197–1024)	384 [196–1663]	0.982
Underlying stent type			0.072
Bare Metal Stent	2 (3.1)	5 (7.8)	
Drug Eluting Stent	48 (75.0)	51 (79.7)	
Bioresorbable vascular scaffold	8 (12.5)	1 (1.6)	
Unknown	6 (9.4)	7 (10.9)	
Ostial lesion	12 (18.8)	17 (26.6)	0.398
Bifurcation lesion	19 (29.7)	25 (39.1)	0.352
Quantitative coronary angiography			
Lesion length, mm	12.4 ± 5.7	14.0 ± 7.4	0.177
Reference vessel diameter, mm	3.0 ± 0.5	2.9 ± 0.5	0.268
Pre-procedural minimal lumen diameter, mm	1.2 ± 0.4	1.1 ± 0.4	0.557
Pre-procedural diameter stenosis, %	60.9 ± 11.5	63.4 ± 13.1	0.256
Post-procedural minimal lumen diameter, mm	2.5 ± 0.5	2.5 ± 0.5	0.863
Post-procedural diameter stenosis, %	19.6 ± 10.3	20.3 ± 7.9	0.673
Predilatation	58 (90.6)	56 (90.3)	1.000
Nominal balloon diameter, mm	3.4 ± 0.5	3.4 ± 0.6	0.966
Maximal balloon pressure, atm	16.0 ± 4.4	15.8 ± 4.4	0.825
Treatment modality			0.267
Drug-coated balloon	38 (59.4)	45 (70.3)	
Drug-eluting stent implantation	26 (40.6)	19 (29.7)	
Maximal stent diameter, mm	3.3 ± 0.5	3.4 ± 0.6	0.300
Total stented length, mm	29.1 ± 14.0	30.4 ± 14.0	0.752
Number of stents	1.2 ± 0.4	1.2 ± 0.4	0.769
Stent type			0.087
Biolimus-eluting stent	1 (1.6)	0 (0.0)	
Everolimus-eluting Stent	25 (39.1)	16 (25.0)	
Paclitaxel-eluting stent	0 (0.0)	1 (1.6)	
Sirolimus-eluting stent	0 (0.0)	2 (3.1)	

Data are shown as counts (%), mean ± SD (standard deviation) or median [25th–75th percentiles]

### Optical coherence tomography analysis

OCT morphometric data according to neointimal tissue characteristics are shown in Table 3. Morphometric analysis included a total of 2315 frames (22,338 struts) in the low inhomogeneity group and 2175 frames (21,191 struts) in the high inhomogeneity group. There were no differences in terms of stent diameter/area, lumen diameter/area or neointimal thickness/area between the groups.

**Table 3:** Optical coherence tomography characteristics according to the extent of inhomogeneity

	Low inhomogeneity *n* = 64	High inhomogeneity *n* = 64	*P* value
Frames analyzed	2315	2175	–
Struts analyzed	22,338	21,191	–
Mean stent area, mm^2^	6.38 (5.04–8.37)	6.44 (4.96–7.83)	0.273
Mean stent diameter, mm	2.85 (2.53–3.26)	2.86 (2.51–3.15)	0.342
Minimal stent diameter, mm	2.70 (2.38–3.09)	2.70 (2.37–2.98)	0.271
Maximal stent diameter, mm	2.99 (2.66–3.45)	3.01 (2.65–3.36)	0.430
Mean lumen area, mm^2^	4.39 (2.93–6.25)	3.96 (2.84–5.85)	0.243
Mean lumen diameter, mm	2.36 (1.92–2.81)	2.24 (1.89–2.72)	0.308
Minimal lumen diameter, mm	2.17 (1.75–2.57)	2.06 (1.71–2.49)	0.317
Maximal lumen diameter, mm	2.56 (2.08–3.08)	2.43 (2.09–2.97)	0.322
Mean area stenosis, %	28.78 (13.97–45.92)	31.18 (15.88–49.08)	0.595
Neointimal area, mm^2^	1.65 (0.90–2.86)	1.88 (1.00–3.03)	0.922
Mean neointimal thickness, μm	170.0 (80.0–320.0)	170.0 (90.0–320.0)	0.396
Strut coverage, %	93.1	93.1	0.113
Strut malapposition, %	0.7	1.3	0.279
Mean malapposition distance, μm	150.0 (130.0–200.0)	180.0 (130.0–300.0)	0.519
Proportion of inhomogeneous quadrants, %	1 (0–5)	20 (12–41)	< 0.001

Data are shown as counts (%) or median (25th–75th percentiles)

OCT morphometric data according to neointimal tissue characteristics are shown in Supplementary Tables 5 and 6 for the subgroups undergoing DCB and DES treatment, respectively, while Supplementary Table 7 shows OCT morphometric data according to the extent of neatherosclerosis in the subgroup of patients with high neointimal inhomogeneity.

### Changes in cardiac biomarkers according to neointimal tissue characteristics and treatment modality

**Fig. 2:**
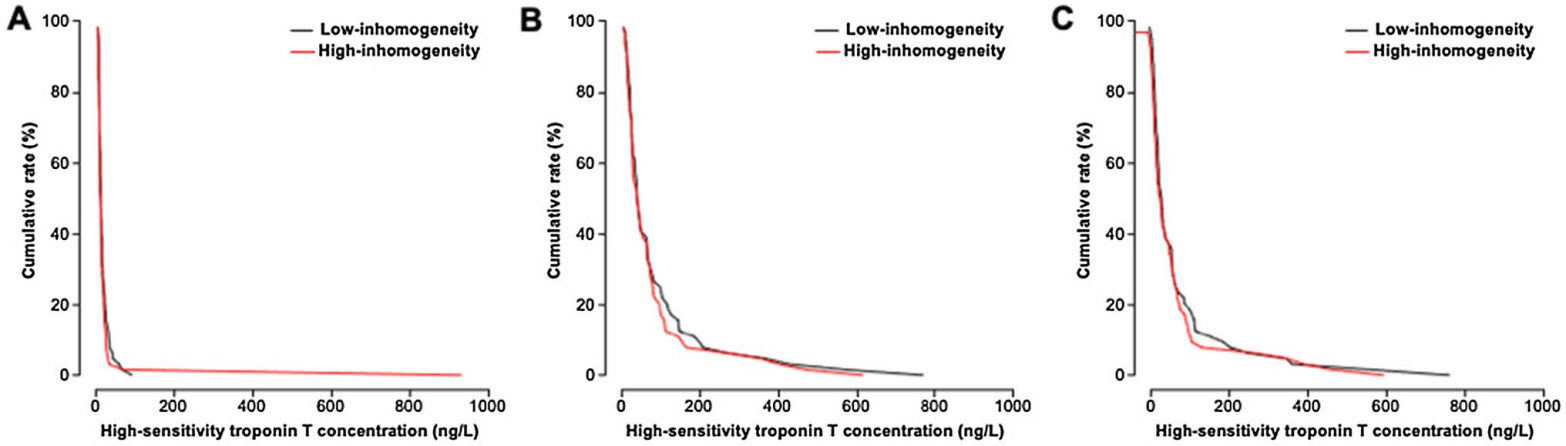
Cumulative frequency distribution curves for baseline **A**, peak post-procedural **B** and delta **C** high-sensitivity cardiac Troponin T concentration

**Fig. 3:**
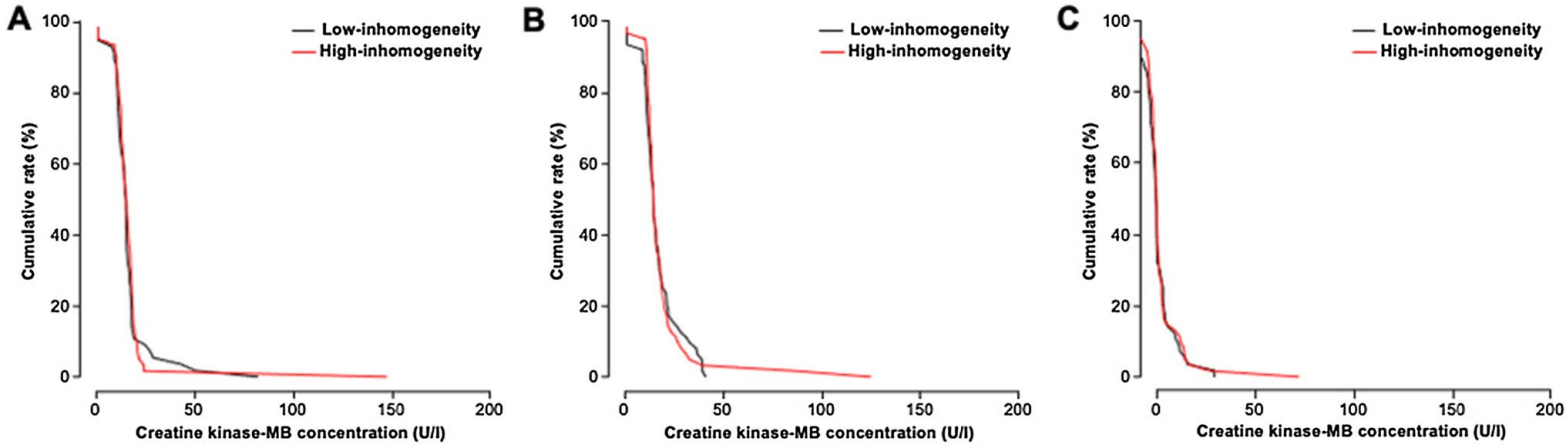
Cumulative frequency distribution curves for baseline (**A**), peak post-procedural **B** and delta **C** creatine kinase-MB concentration

Table 4 shows changes in hs-cTnT and CK-MB as well as the incidence of minor and major PMI according to the prevalence of inhomogeneous quadrants and/or treatment modality (DCB vs. DES). There were no significant differences in terms of changes in hs-cTnT or CK-MB levels, or minor/major PMI incidence, neither according to prevalence of inhomogeneous quadrants, nor to treatment modality. Even after adjusting for potential confounders, type of neointimal tissue did not independently correlate with changes in hs-cTnT (*p* = 0.468). Additionally, no significant interaction was present between optical neointimal characteristics and treatment modality in terms of changes in hs-cTnT (*P*_int_ = 0.432). Cumulative frequency distribution curves for baseline, peak post-procedural and changes in hs-cTnT and CK-MB in the low and high inhomogeneity groups are shown in Figs. 2, 3. Finally, no significant differences in terms of peak values or changes in hs-cTnT or CK-MB were detected according to the extent of neatherosclerosis in the subgroup of patients with high neointimal inhomogeneity.

**Table 4:** Biomarker values according to neointimal tissue characterization and/or treatment modality

Cardiac biomarker values according to neointimal tissue characterization			
	Low inhomogeneity *N* = 64	High inhomogeneity *N* = 64	*p* value
Baseline hs-cTnT, ng/L	10.0 (7.0–18.2)	11.5 (8.0–18.0)	0.697
Peak post-procedural hs-cTnT, ng/L	40.5 (23.5–99.8)	40.5 (23.2–80.2)	0.728
Delta hs-cTnT, ng/L	28.0 (12.0–65.8)	25.5 (9.8–65.0)	0.355
Major PMI	20 (31.2%)	20 (31.2%)	1.000
Minor PMI	62 (96.9%)	62 (96.9%)	1.000
Baseline CK-MB, U/l	14.9 (11.2–17.4)	15.3 (12.4–18.0)	0.416
Peak post-procedural CK-MB, U/l	14.5 (11.2–20.1)	14.4 (12.3–18.7)	0.684
Delta CK-MB, U/l	− 0.2 (− 2.9–3.4)	− 0.1 (− 1.80–2.6)	0.562

Cardiac biomarker values according to treatment modality			
	Drug-coated balloon *N* = 83	Drug-eluting stent *N* = 45	
Baseline hs-cTnT, ng/L	10.0 (7.0–18.5)	12.0 (8.0–18.0)	0.288
Peak post-procedural hs-cTnT, ng/L	39.0 (22.5–79.0)	46.0 (24.0–99.0)	0.445
Delta hs-cTnT, ng/L	27.0 (10.0–64.0)	28.0 (11.0–73.0)	0.795
Major PMI	24 (28.9%)	16 (35.6%)	0.566
Minor PMI	80 (96.4%)	44 (97.8%)	1.000
Baseline CK-MB, U/l	15.2 (11.6–17.7)	14.5 (11.7–17.9)	0.853
Peak post-procedural CK-MB, U/l	14.4 (12.3–18.3)	14.5 (11.4–21.4)	0.882
Delta CK-MB, U/l	0.0 (− 1.8–2.8)	− 0.6 (− 2.7–3.0)	0.653

Cardiac biomarker values according to neointimal tissue characterization in the subgroup treated with drug-coated balloon			
	Low inhomogeneity *N* = 38	High inhomogeneity *N* = 45	*p *value
Baseline hs-cTnT, ng/L	9.5 (7.0–23.5)	10.0 (7.0–14.0)	0.985
Peak post-procedural hs-cTnT, ng/L	44.0 (26.5–113.0)	30.0 (20.0–66.0)	0.075
Delta hs-cTnT, ng/L	31.0 (18.2–82.2)	18.0 (9.0–55.0)	0.031
Major PMI	13 (34.2%)	11 (24.4%)	0.462
Minor PMI	37 (97.4%)	43 (95.6%)	1.000
Baseline CK-MB, U/l	15.2 (11.7–17.5)	15.3 (11.9–17.8)	0.936
Peak post-procedural CK-MB, U/l	14.6 (12.1–20.6)	14.4 (12.4–17.5)	0.731
Delta CK-MB, U/l	0.0 (− 2.6–3.4)	− 0.10 (− 1.7–2.3)	0.842

Cardiac biomarker values according to neointimal tissue characterization in the subgroup treated with drug-eluting stent			
	Low inhomogeneity *N* = 26	High inhomogeneity *N* = 19	*p* value
Baseline hs-cTnT, ng/L	11.5 (8.0–15.5)	13.0 (8.5–21.5)	0.295
Peak post-procedural hs-cTnT, ng/L	33.5 (22.0.73.0)	73.0 (33.0–130.0)	0.061
Delta hs-cTnT, ng/L	19.0 (11.0–56.2)	48.0 (17.5–96.0)	0.215
Major PMI	7 (26.9%)	9 (47.4%)	0.271
Minor PMI	25 (96.2%)	19 (100%)	1.000
Baseline CK-MB, U/l	14.5 (11.0–15.4)	15.4 (12.7–20.2)	0.278
Peak post-procedural CK-MB, U/l	13.6 (10.9–18.9)	16.2 (11.7–22.9)	0.242
Delta CK-MB, U/l	− 1.0 (−4.1–2.2)	0.2 (− 1.9–3.2)	0.304

Cardiac biomarker values in the subgroup with high neointimal inhomogeneity, according to the extent of neoatherosclerosis			
	Low neoatherosclerosis *N* = 33	High neoatherosclerosis *N* = 31	*p* value
Baseline hs-cTnT, ng/L	12.0 (8.0–19.0)	9.0 (6.0–14.0)	0.049
Peak post procedural hs-cTnT, ng/L	33.0 (26.0–97.0)	47.0 (22.5–75.5)	0.989
Delta hs-cTnT, ng/L	15.0 (6.0–73.0)	31.0 (14.5–57.5)	0.295
Major PMI	10 (30.3%)	10 (32.3%)	1.000
Minor PMI	31 (93.9%)	31 (100%)	0.493
Baseline CK-MB, U/l	15.3 (12.4–17.4)	15.2 (12.5–18.6)	0.697
Peak post-procedural CK-MB, U/l	14.5 (12.4–19.3)	14.4 (11.6–18.3)	0.693
Delta CK-MB, U/l	− 0.1 (− 1.8–3.4)	− 0.0 (− 1.7–1.2)	0.673

Data are shown as counts (%) or median [25th–75th percentiles]

### Clinical outcomes according to optical characteristics of the neointima

**Fig. 4:**
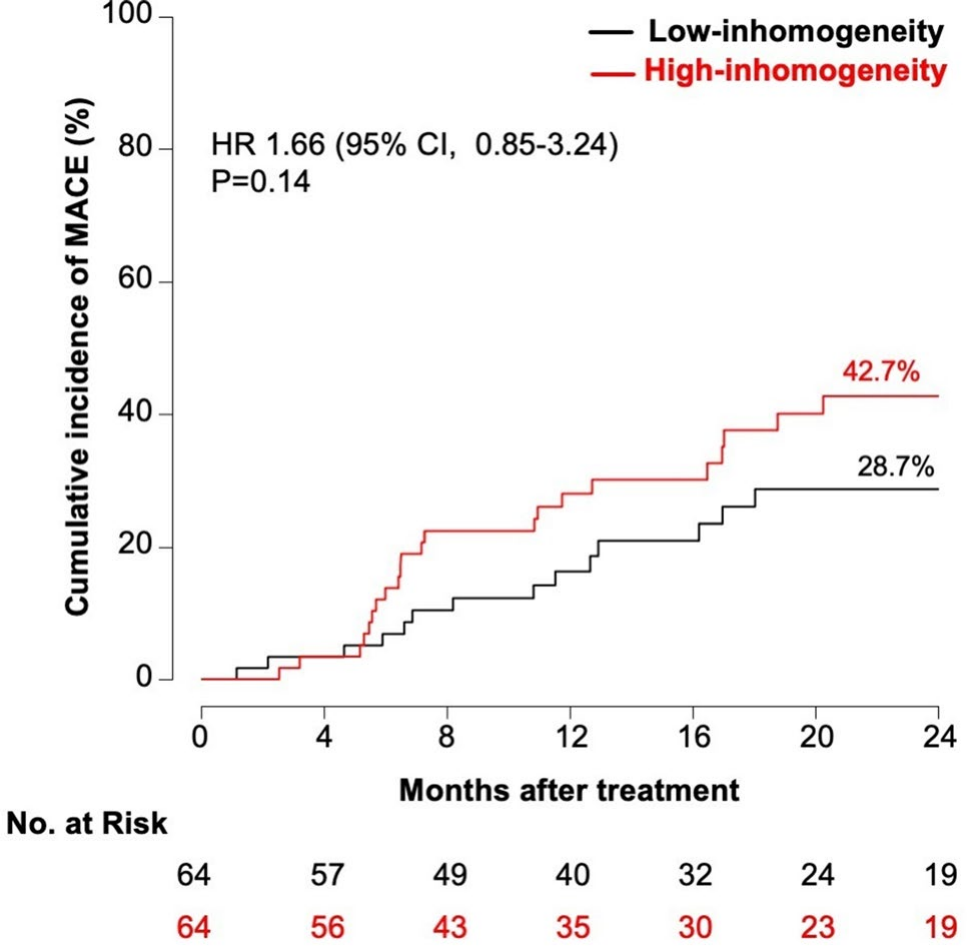
Two-year cumulative incidence of major adverse cardiac events according to neointimal tissue characterization *CI* confidence interval, *HR *hazard ratio, *MACE* major adverse cardiac events

**Fig. 5:**
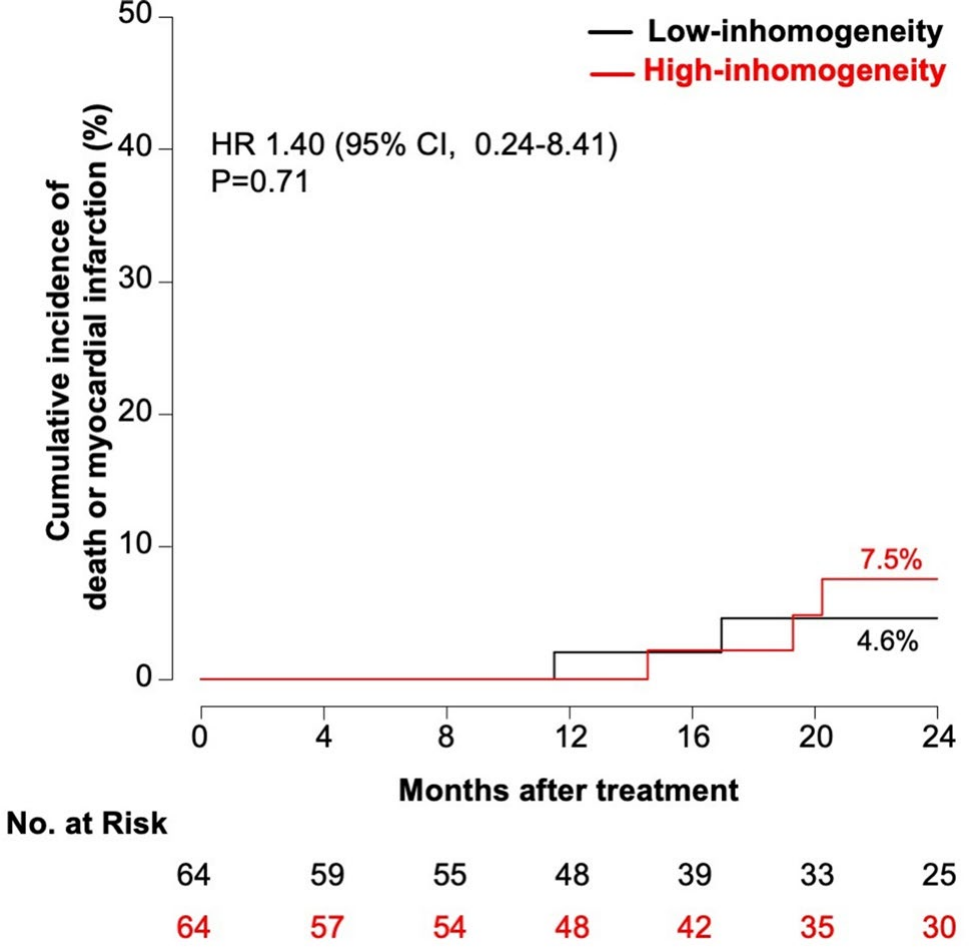
Two-year cumulative incidence of death or myocardial infarction according to neointimal tissue characterization *CI* confidence interval, *HR* hazard ratio, *MI* myocardial infarction

**Fig. 6:**
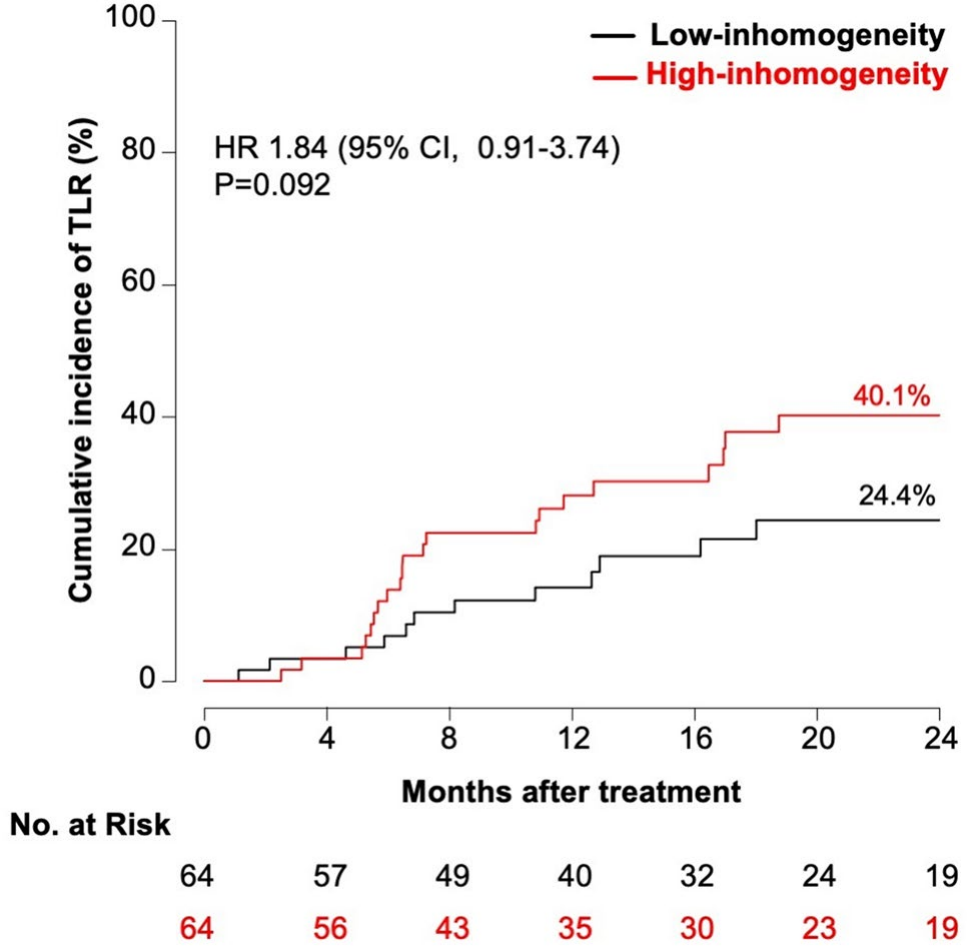
Two-year cumulative incidence of target-lesion revascularization according to neointimal tissue characterization *CI* confidence interval, *HR* hazard ratio, *TLR* target-lesion revascularization

There were no significant differences in terms of MACE (42.7 vs. 28.7%; HR 1.66 [95% CI, 0.85–3.24], p = 0.14) (Fig. 4), composite of death or MI (7.5 vs. 4.6%; HR 1.40 [95% CI, 0.24–8.41], *p* = 0.71) (Fig. 5), or clinically driven TLR (40.1 vs. 24.4%; HR 1.84 [95% CI, 0.91–3.74], *p* = 0.092) (Fig. 6) between the groups displaying low and high neointimal inhomogeneity.

## Discussion

The key findings of this report can be summarized as follows: (1) the incidence of PMI following PCI for ISR is high and broadly comparable to the incidence of PMI following native vessel PCI; (2) there was no association between increasing neointimal inhomogeneity and PMI occurrence; (3) within the high neointimal inhomogeneity subgroup, increasing expression of neoatherosclerotic changes did not impact the occurrence of PMI; (4) PMI occurrence was not influenced by treatment modality (DCB or DES) of ISR lesions.

PMI has been reported in a high proportion of patients following otherwise uneventful PCI procedures for stable CAD [4–8]. However, the reported incidence of PMI varies significantly depending on cardiac biomarker and definition used; moreover, due to discordant study results [4, 5, 7, 8, 10], the prognostic relevance of PMI represents an object of ongoing controversy and the cut-off thresholds for defining PMI have been mostly based on expert consensus opinions [6, 19]. Based on the results of a recent patient-level pooled analysis (PMI incidence according to 4th UDMI of 52.8 and 79.8% if restricted to hs-cTn) [8], a recent consensus document subdivided PMI in prognostically relevant, “major” PMI and “minor” PMI [9]. Applying such definition, the incidence of hs-cTnT-based major PMI in the present study was ≈30%, thereby confirming the relevant occurrence of PMI not only in native vessel but also in ISR-PCI.

The number of studies investigating neointimal characteristics and PMI occurrence in the setting of PCI for ISR is extremely limited [13, 14]. Kimura et al. [14] evaluated the relationship between PMI occurrence (defined as hs-cTnT > 5 × 99th percentile URL) and neointimal tissue characteristics evaluated by means of OCT and coronary angioscopy in 72 patients undergoing ISR-PCI. The authors reported thinner fibrous cap and a higher presence of thin-cap fibroatheroma in lesions with PMI as compared to those without. However, at multivariate analysis only atheromatous appearance at coronary angioscopy independently correlated with PMI. Lee et al. [13] investigated the relationship between PMI occurrence (defined as CK-MB > 99th percentile URL) and optical characteristics of neointima in 125 patients undergoing PCI for ISR. The authors found a significant association between increased expression of neoatherosclerotic changes and thin-cap fibroatheroma and the occurrence of PMI. In the present study, which combined a detailed, quadrant-based multi-frame neointimal characterization coupled to systematic pre- and post-procedural hs-cTnT measurements, we did not observe any significant differences in terms of major or minor PMI occurrence between patients with high versus those with low neointimal inhomogeneity. Moreover, an increasing expression of neoatherosclerotic changes had no impact on PMI occurrence.

An important finding of the present study was the absence of significant differences in PMI occurrence following treatment of ISR with DCB as compared with treatment with DES. Indeed, DCB angioplasty might be associated with a risk of distal embolization of particulate balloon coating, consisting of antirestenotic drug and excipient [17]. Examination of downstream microvascular beds in preclinical studies has occasionally revealed distal embolization of microparticles of matrix coating. The findings of the present study, which confirm those of a previous report from our group [20], speak against a relevant difference in subclinical myocardial injury and support the safety of DCB use for the treatment of ISR.

Some limitations of the present study should be mentioned. First, this was a single-center, moderate-sized, retrospective study and selection bias might have occurred in the decision to perform intravascular imaging. Second, treatment modality of ISR was at the discretion of the operator and consequently additional selection bias might have been introduced at this stage. Third, this study was not powered to detect differences in mortality according to the presence or absence of PMI.

## Conclusions

In patients undergoing DCB or DES treatment of ISR lesions, the incidence of PMI is high and broadly comparable to PMI incidence following native vessel PCI. There was no association between increasing neointimal inhomogeneity, or increasing expression of neoatherosclerotic changes, and occurrence of PMI. Despite evidence of distal particulate embolization of DCB matrix coating in preclinical studies, the treatment modality of ISR (DCB- or DES-based) did not appear to impact the occurrence of PMI, thereby supporting the safety of DCB angioplasty for the treatment of ISR.

## Supplementary Information

Below is the link to the electronic supplementary material.

Supplementary file1 (DOCX 48 KB)

## Abbreviations

CK-MB: Creatine kinase myocardial band
DCB: Drug-coated balloon
DES: Drug-eluting stent
hs-cTnT: High-sensitivity cardiac troponin T
ISR: N-stent restenosis
OCT: Optical coherence tomography
PMI: Periprocedural myocardial injury
UDMI: Universal definition of myocardial infarction
