# Supplementary material for: Periprocedural myocardial injury according to optical characteristics of neointima and treatment modality of in-stent restenosis
Source: Clin Res Cardiol. 2022 Apr 27;111(7):827–37. doi: 10.1007/s00392-022-02024-z (PMC9242953; doi:10.1007/s00392-022-02024-z)
Supplement: Supplementary file 1 — Supplementary file1 (DOCX 48 KB) [file 392_2022_2024_MOESM1_ESM.docx]

**SUPPLEMENTARY APPENDIX TO:**

**Periprocedural myocardial injury according to optical characteristics of neointima and treatment modality of in-stent restenosis**

Nejva Nano, MD^1^; Alp Aytekin, MD^1^; Gjin Ndrepepa^,^ MD^1^; Masaru Seguchi, MD^1^; Jola Bresha, MD^2;^ Hector Alfonso Alvarez Covarrubias, MD^1^; Philipp Nicol, MD^1^; Tobias Lenz, MD^1^; Shqipdona Lahu, MD^1^; Senta Gewalt, MD^1^; Felix Voll, MD^1^; Tobias Rheude, MD^1^; Jens Wiebe, MD^1^; Heribert Schunkert, MD^1,3^; Sebastian Kufner^,^ MD^1^; Salvatore Cassese, MD, PhD^1^; Michael Joner, MD ^1,3^; Adnan Kastrati, MD^1,3^; Erion Xhepa, MD, PhD^1^

^1^Klinik für Herz- und Kreislauferkrankungen, Deutsches Herzzentrum München, Technische Universität München, Munich, Germany

^2^Medizinische Klinik und Poliklinik Innere Medizin I, Klinikum rechts der Isar, Technische Universität München, Munich, Germany

^3^DZHK (German Centre for Cardiovascular Research), partner site Munich Heart Alliance, Munich, Germany

**Supplementary Main text**

*Angiographic data acquisition and analysis*

Baseline and post-procedural angiograms were recorded and assessed off-line in a core laboratory (ISAResearch Center, Munich, Germany) with an automated edge-detection system (Medis Medical Imaging Systems, Leiden, The Netherlands). Measurements were performed on cineangiograms recorded after intracoronary administration of nitroglycerine. The contrast-filled, non-tapered catheter tip was used for calibration. Quantitative analysis was performed on both “in-stent” and in-segment” areas (including the stented segment as well both 5 mm margins proximal and distal to the stent).

*OCT data acquisition and analysis*

Following administration of intracoronary nitrates, OCT was performed with non-occlusive imaging technique using commercially-available OCT imaging systems (C7XR, Ilumien or Ilumien Optis, St. Jude Medical, St. Paul, MN, USA). In brief, a rapid exchange imaging catheter (Dragonfly^TM^ or Dragonfly Duo^TM^, St. Jude Medical, St. Paul, MN, USA) was advanced beyond the stented segment. An OCT pullback of the entire stented segment, including distal and proximal reference sites, was performed with contrast injection through the guiding catheter at 3-5 ml/sec. If the stented segment was too long to be imaged in a single pullback, an additional pullback was acquired using angiographic landmarks for appropriate imaging catheter position and view. In case of sub-occlusive or occlusive ISR lesions, small balloon dilatation (≤2.0 mm in diameter) at low pressure was performed to allow sufficient blood clearance and pullback quality.

Raw data of OCT image acquisitions were sent to a centralized core laboratory (ISAResearch Center, Munich, Germany) for off-line analyses. Quantitative and morphometric analyses were performed every 1 mm along the entire target segment by means of dedicated software (St. Jude Medical, St. Paul, MN, USA).

The first and last analyzed frames of the stented segment were defined as OCT frames where stent struts were present in at least ¾ of the perimeter. Stent and lumen cross-sectional area were measured throughout the entire length of the stent. The number of stent struts was recorded for each analyzed cross-section. Thickness of tissue coverage on the luminal side of each stent strut was measured at the midpoint of the strut. Struts were classified as covered if the thickness of tissue covering the strut was ≥ the minimal axial resolution of OCT (20 μm). Struts were considered uncovered if any part of the strut was visibly exposed to the lumen. Incomplete stent strut apposition was considered present when the axial distance between the strut’s surface to the luminal surface was > the sum of the strut and polymer thickness plus the minimal axial resolution of OCT.

Distal and proximal reference measurements were performed in none or minimally diseased cross-sections within 10 mm from the stent edges. The reference area was calculated as the sum of the proximal and distal reference lumen area divided by 2. If the pullback did not include analyzable proximal and/or distal non-stented reference segments, the reference area was derived from the most proximal and/or distal stented segments.

*Qualitative neointimal characterization*

Since previous studies have shown considerable intralesion neointimal heterogeneity, characterization of neointimal tissue was performed not only at the frame displaying the maximal % area stenosis (%AS), but also in correspondence of the 5 preceding and following analyzed frames. Each frame was subdivided in 4 quadrants (90°) and the neointimal characteristics separately characterized for each of them. Previous validation studies against the gold standard of histology have shown homogeneous patterns to consistently correlate with abundance of smooth muscle cells embedded in collagen/proteoglycan rich tissue, while the remaining patterns revealed a multitude of corresponding histological components. Therefore, in order to apply a histopathology-based and treatment-oriented classification, neointimal tissue was categorized as homogeneous or inhomogeneous, the latter category including heterogeneous, layered or neoatherosclerosis quadrants. Atherosclerotic changes of the neointima were defined by the presence of one or more of the following: macrophage infiltration, lipid-laden tissue within the stent or neointimal calcification.

**Supplementary Tables**

**Supplementary Table S1*.* Clinical characteristics according to treatment modality of in-stent restenosis**

|  | Drug-coated balloon  N=83 | Drug-eluting stent  N=45 | p-value |
| --- | --- | --- | --- |
| Age, years | 67.5±9.8 | 67.7±10.0 | 0.945 |
| Sex, male | 17 (20.5) | 6 (13.3) | 0.444 |
| Body mass index, kg/ m^2^ | 28.5 (4.1) | 28.4 (4.3) | 0.952 |
| Current smoker | 12 (14.5) | 11 (24.4) | 0.244 |
| Ex-Smoker | 29 (34.9) | 14 (31.1) | 0.809 |
| Hypercholesterolemia | 53 (63.9) | 34 (75.6) | 0.248 |
| Arterial hypertension | 81 (97.6) | 43 (95.6) | 0.612 |
| Diabetes mellitus | 40 (48.2) | 17 (37.8) | 0.344 |
| Oral therapy | 18 (21.7) | 14 (31.1) | 0.336 |
| Insulin therapy | 15 (18.1) | 2 (4.4) | 0.058 |
| Previous coronary artery bypass surgery | 15 (18.1) | 7 (15.6) | 0.908 |
| Previous myocardial infarction | 42 (50.6) | 22 (48.9) | 1.000 |
| *Clinical presentation* |  |  | 0.600 |
| Silent Ischemia | 20 (24.1) | 10 (22.2) |  |
| Stable Angina Pectoris | 51 (61.4) | 31 (68.9) |  |
| Unstable Angina Pectoris | 12 (14.5) | 4 (8.9) |  |
| *Number of diseased coronary arteries* |  |  | 0.876 |
| One vessel | 9 (10.8) | 5 (11.1) |  |
| Two vessels | 14 (16.9) | 9 (20.0) |  |
| Three vessels | 60 (72.3) | 31 (68.9) |  |
| Multi-vessel disease | 74 (89.2) | 40 (88.9) | 1.000 |
| Left ventricular ejection fraction, % | 53.2±8.7 | 49.5±13.3 | 0.367 |

Data are shown as counts (%) or mean±SD (standard deviation)

**Supplementary Table S2. Angiographic and procedural characteristics according to treatment modality of in-stent restenosis**

|  | Drug-coated balloon  N= 83 | Drug-eluting stent  N= 45 | p-value |
| --- | --- | --- | --- |
| *Target vessel* |  |  | 0.050 |
| Left main coronary artery | 2 (2.4) | 1 (2.2) |  |
| Left anterior descending artery | 33 (39.8) | 27 (60.0) |  |
| Left circumflex artery | 25 (30.1) | 5 (11.1) |  |
| Right coronary artery | 23 (27.7) | 12 (26.7) |  |
| *Restenosis morphology* |  |  | 0.449 |
| Focal margin | 5 (6.0) | 2 (4.4) |  |
| Focal body | 43 (51.8) | 19 (42.2) |  |
| Multifocal | 8 (9.6) | 4 (8.9) |  |
| Diffuse intrastent | 25 (30.1) | 16 (35.6) |  |
| Proliferative | 0 (0.0) | 2 (4.4) |  |
| Complete occlusion | 2 (2.4) | 2 (4.4) |  |
| Index stent interval, days | 295 [197-1030] | 428 [202-2017] | 0.277 |
| *Underlying stent type* |  |  | <0.001 |
| Bare Metal Stent | 3 (3.6) | 4 (8.9) |  |
| Drug Eluting Stent | 74 (89.2) | 25 (55.6) |  |
| Bioresorbable vascular scaffold | 1 (1.2) | 8 (17.8) |  |
| Unknown | 5 (6.0) | 8 (17.8) |  |
| Ostial lesion | 24 (28.9) | 5 (11.1) | 0.038 |
| Bifurcation lesion | 33 (39.8) | 11 (24.4) | 0.122 |
| *Quantitative coronary angiography* | | | |
| Lesion length, mm | 12.1±5.6 | 15.1±7.9 | 0.027 |
| Reference vessel diameter, mm | 3.0±0.5 | 3.0±0.5 | 0.926 |
| Pre-procedural minimal lumen diameter, mm | 1.2±0.4 | 1.1±0.4 | 0.733 |
| Pre-procedural diameter stenosis, % | 61.7±12.1 | 62.9±12.8 | 0.629 |
| Post-procedural minimal lumen diameter, mm | 2.3±0.4 | 2.8±0.5 | <0.001 |
| Post-procedural diameter stenosis, % | 24.3±7.5 | 12.1±6.1 | <0.001 |
| Predilatation | 76 (92.7) | 38 (86.4) | 0.340 |
| Nominal balloon diameter, mm | 3.3±0.4 | 3.5±0.7 | 0.046 |
| Maximal balloon pressure, atm | 15.0±4.4 | 17.6±4.0 | 0.001 |

Data are shown as counts (%), mean±SD (standard deviation) or median [25^th^-75th percentiles]

**Supplementary Table S3. Clinical characteristics of the subgroup with high neointimal inhomogeneity according to the extent of neoatherosclerosis**

|  | Low neoatherosclerosis  N=33 | High neoatherosclerosis  N=31 | p-value |
| --- | --- | --- | --- |
| Age, years | 68.4±7.0 | 68.3±10.6 | 0.987 |
| Sex, male | 6 (18.2) | 7 (22.6) | 0.900 |
| Body mass index, kg/ m^2^ | 29.1±3.9 | 27.6±5.1 | 0.184 |
| Current smoker | 4 (12.1) | 6 (19.4) | 0.504 |
| Ex-Smoker | 12 (36.4) | 11 (35.5) | 1.000 |
| Hypercholesterolemia | 22 (66.7) | 22 (71.0) | 0.919 |
| Arterial hypertension | 33 (100.0) | 30 (96.8) | 0.484 |
| Diabetes mellitus | 14 (42.4) | 14 (45.2) | 1.000 |
| Oral therapy | 5 (15.2) | 11 (35.5) | 0.112 |
| Insulin therapy | 5 (15.2) | 1 (3.2) | 0.198 |
| Previous coronary artery bypass surgery | 8 (24.2) | 4 (12.9) | 0.400 |
| Previous myocardial infarction | 19 (57.6) | 11 (35.5) | 0.129 |
| *Clinical presentation* |  |  | 0.184 |
| Silent Ischemia | 10 (30.3) | 4 (12.9) |  |
| Stable Angina Pectoris | 20 (60.6) | 25 (80.6) |  |
| Unstable Angina Pectoris | 3 (9.1) | 2 (6.5) |  |
| *Number of diseased coronary arteries* |  |  | 0.385 |
| One vessel | 6 (18.2) | 2 (6.5) |  |
| Two vessels | 7 (21.2) | 6 (19.4) |  |
| Three vessels | 20 (60.6) | 23 (74.2) |  |
| Multi-vessel disease | 27 (81.8) | 29 (93.5) | 0.259 |
| Left ventricular ejection fraction, % | 51.3±11.2 | 52.7±9.5 | 0.723 |

Data are shown as counts (%) or mean±SD (standard deviation)

**Supplementary Table S4. Angiographic and procedural characteristics of the subgroup with high neointimal inhomogeneity according to the extent of neoatherosclerosis**

|  | Low neoatherosclerosis  N= 33 | High neoatherosclerosis  N= 31 | p-value |
| --- | --- | --- | --- |
| *Target vessel* |  |  | 0.702 |
| Left main coronary artery | 2 (6.1) | 1 (3.2) |  |
| Left anterior descending artery | 17 (51.5) | 13 (41.9) |  |
| Left circumflex artery | 8 (24.2) | 11 (35.5) |  |
| Right coronary artery | 6 (18.2) | 6 (19.4) |  |
| *Restenosis morphology* |  |  | 1.000 |
| Focal margin | 3 (9.1) | 2 (6.5) |  |
| Focal body | 15 (45.5) | 15 (48.4) |  |
| Multifocal | 1 (3.0) | 1 (3.2) |  |
| Diffuse intrastent | 12 (36.4) | 11 (35.5) |  |
| Proliferative | 1 (3.0) | 0 (0.0) |  |
| Complete occlusion | 1 (3.0) | 2 (6.5) |  |
| Index stent interval, days | 295 [189-847] | 479 [209-1751] | 0.196 |
| *Underlying stent type* |  |  | 0.805 |
| Bare Metal Stent | 2 (6.1) | 3 (9.7) |  |
| Drug Eluting Stent | 27 (81.8) | 24 (77.4) |  |
| Bioresorbable vascular scaffold | 0 (0.0) | 1 (3.2) |  |
| Unknown | 4 (12.1) | 3 (9.7) |  |
| Ostial lesion | 9 (27.3) | 8 (25.8) | 1.000 |
| Bifurcation lesion | 13 (39.4) | 12 (38.7) | 1.000 |
| *Quantitative coronary angiography* | | | |
| Lesion length, mm | 13.7±6.7 | 14.2±8.19 | 0.785 |
| Reference vessel diameter, mm | 2.9±0.5 | 2.9±0.6 | 0.635 |
| Pre-procedural minimal lumen diameter, mm | 1.1±0.4 | 1.1±0.4 | 0.791 |
| Pre-procedural diameter stenosis, % | 64.3±12.7 | 62.5±13.6 | 0.598 |
| Post-procedural minimal lumen diameter, mm | 2.4±0.4 | 2.5±0.6 | 0.734 |
| Post-procedural diameter stenosis, % | 21.2±7.2 | 19.3±8.5 | 0.363 |
| Predilatation | 30 (93.8) | 26 (86.7) | 0.418 |
| Nominal balloon diameter, mm | 3.4±0.5 | 3.3±0.6 | 0.425 |
| Maximal balloon pressure, atm | 15.8±4.5 | 15.9±4.3 | 0.940 |
| *Treatment modality* |  |  | 0.071 |
| Drug-coated balloon | 27 (81.8) | 18 (58.1) |  |
| Drug-eluting stent implantation | 6 (18.2) | 13 (41.9) |  |
| Maximal stent diameter, mm | 3.5±0.4 | 3.4±0.7 | 0.335 |
| Total stented length, mm | 33.0±16.8 | 29.2±13.1 | 0.640 |
| Number of Stents | 1.3±0.5 | 1.1±0.3 | 0.294 |
| *Stent type* |  |  | 0.070 |
| Everolimus-eluting Stent | 6 (18.2) | 10 (32.3) |  |
| Paclitaxel-eluting stent | 0 (0.0) | 1 (3.2) |  |
| Sirolimus-eluting stent | 0 (0.0) | 2 (6.5) |  |

Data are shown as counts (%), mean±SD (standard deviation) or median [25^th^; 75th percentiles]

**Supplementary Table S5*.* Optical coherence tomography characteristics according to neointimal tissue characterization in the subgroup treated with drug-coated balloon**

|  | Low inhomogeneity N=38 | High inhomogeneity N=45 | P-value |
| --- | --- | --- | --- |
| Frames analyzed | 1463 | 1683 | - |
| Struts analyzed | 14605 | 16898 | - |
| Mean stent area, mm^2^ | 6.48 (5.05-8.72) | 6.64 (5.14-8.10) | 0.577 |
| Mean stent diameter, mm | 2.87 (2.53-3.32) | 2.90 (2.55-3.20) | 0.718 |
| Minimal stent diameter, mm | 2.74 (2.38-3.15) | 2.74 (2.41-3.02) | 0.712 |
| Maximal stent diameter, mm | 3.05 (2.66-3.54) | 3.06 (2.68-3.44) | 0.723 |
| Mean lumen area, mm^2^ | 4.40 (2.88-6.44) | 4.14 (2.97-6.06) | 0.508 |
| Mean lumen diameter, mm | 2.36 (1.91-2.85) | 2.29 (1.93-2.77) | 0.643 |
| Minimal lumen diameter, mm | 2.15 (1.74-2.62) | 2.10 (1.75-2.54) | 0.706 |
| Maximal lumen diameter, mm | 2.58 (2.05-3.12) | 2.48 (2.13-3.02) | 0.598 |
| Mean area stenosis, % | 30.25 (15.03-46.27) | 29.91 (15.36-47.76) | 0.834 |
| Neointimal area, mm^2^ | 1.73 (0.95-3.11) | 1.84 (1.00-3.08) | 0.889 |
| Mean neointimal thickness, μm | 170.0 (80.0-320.0) | 170.0 (80.0-300.0) | 0.856 |
| Strut coverage, % | 92.8 | 92.0 | 0.450 |
| Strut malapposition, % | 0.9 | 1.5 | 0.349 |
| Mean malapposition distance, μm | 150.0 (130.0-210.0) | 180.0 (130.0-320.0) | 0.641 |

Data are shown as counts (%) or median (25^th^-75^th^ percentiles)

**Supplementary Table S6*.* Optical coherence tomography characteristics according to neointimal tissue characterization in the subgroup treated with drug-eluting stent**

|  | Low inhomogeneity N=26 | High inhomogeneity N=19 | P-value |
| --- | --- | --- | --- |
| Frames analyzed | 852 | 492 | - |
| Struts analyzed | 7733 | 4293 | - |
| Mean stent area, mm^2^ | 6.17 (5.03-7.91) | 5.85 (4.50-7.08) | 0.117 |
| Mean stent diameter, mm | 2.80 (2.52-3.17) | 2.73 (2.39-3.00) | 0.128 |
| Minimal stent diameter, mm | 2.66 (2.37-2.99) | 2.59 (2.24-2.85) | 0.093 |
| Maximal stent diameter, mm | 2.92 (2.65-3.33) | 2.87 (2.52-3.15) | 0.186 |
| Mean lumen area, mm^2^ | 4.38 (3.00-5.98) | 3.57 (2.34-4.9) | 0.189 |
| Mean lumen diameter, mm | 2.36 (1.95-2.75) | 2.11 (1.71-2.50) | 0.199 |
| Minimal lumen diameter, mm | 2.19 (1.76-2.53) | 1.96 (1.57-2.32) | 0.193 |
| Maximal lumen diameter, mm | 2.54 (2.14-2.94) | 2.30 (1.88-2.76) | 0.223 |
| Mean area stenosis, % | 26.02 (12.97-44.03) | 35.57 (18.7-54.55) | 0.584 |
| Neointimal area, mm^2^ | 1.56 (0.78-2.64) | 1.96 (0.99-2.94) | 0.760 |
| Mean neointimal thickness, μm | 170.0 (80.0-310.0) | 210.0 (110.0-410.0) | 0.050 |
| Strut coverage, % | 93.6 | 97.6 | <0.001 |
| Strut malapposition, % | 0.4 | 0.7 | 0.791 |
| Mean malapposition distance, μm | 150.0 (130.0-190.0) | 200.0 (140.0-240.0) | 0.757 |

Data are shown as counts (%) or median (25^th^-75^th^ percentiles)

**Supplementary Table S7*.* Optical coherence tomography characteristics of the subgroup with high neointimal inhomogeneity according to extent of neoatherosclerosis**

|  | Low inhomogeneity N=33 | High inhomogeneity N=31 | P-value |
| --- | --- | --- | --- |
| Frames analyzed | 1209 | 966 | - |
| Struts analyzed | 11863 | 9328 | - |
| Mean stent area, mm^2^ | 6.45 (4.91-7.88) | 6.41 (5.05-7.76) | 0.808 |
| Mean stent diameter, mm | 2.86 (2.50-3.16) | 2.85 (2.54-3.14) | 0.817 |
| Minimal stent diameter, mm | 2.70 (2.35-2.98) | 2.70 (2.39-2.98) | 0.923 |
| Maximal stent diameter, mm | 3.00 (2.64-3.39) | 3.01 (2.67-3.35) | 0.733 |
| Mean lumen area, mm^2^ | 4.20 (2.98-6.09) | 3.71 (2.71-5.55) | 0.197 |
| Mean lumen diameter, mm | 2.30 (1.94-2.78) | 2.17 (1.85-2.65) | 0.192 |
| Minimal lumen diameter, mm | 2.13 (1.76-2.57) | 2.00 (1.66-2.39) | 0.160 |
| Maximal lumen diameter, mm | 2.49 (2.13-3.01) | 2.36 (2.01-2.91) | 0.240 |
| Mean area stenosis, % | 26.34 (14.97-44.69) | 37.28 (19.38-53.44) | 0.168 |
| Neointimal area, mm^2^ | 1.67 (0.91-2.60) | 2.18 (1.12-3.56) | 0.115 |
| Mean neointimal thickness, μm | 160.0 (80.0-290.0) | 200.0 (100.0-360.0) | 0.072 |
| Strut coverage, % | 92.2 | 94.4 | 0.282 |
| Strut malapposition, % | 1.4 | 1.3 | 0.995 |
| Mean malapposition distance, μm | 160.0 (130.0-280.0) | 220.0 (150.0-335.0) | 0.890 |

Data are shown as counts (%) or median (25^th^-75^th^ percentiles)
